# Correction: Model Cortical Association Fields Account for the Time Course and Dependence on Target Complexity of Human Contour Perception

**DOI:** 10.1371/annotation/35890214-d064-4b76-8bfc-5ac1ab07c8b8

**Published:** 2011-10-25

**Authors:** Vadas Gintautas, Michael I. Ham, Benjamin Kunsberg, Shawn Barr, Steven P. Brumby, Craig Rasmussen, John S. George, Ilya Nemenman, Luís M. A. Bettencourt, Garrett T. Kenyon

The tenth author's name was spelled incorrectly. The correct name is: Garrett T. Kenyon.

This error was introduced in the preparation of this article for publication.

